# Traumatic Iridodialysis Associated With Hyphema Secondary to Injury From a Bungee Cord

**DOI:** 10.7759/cureus.13280

**Published:** 2021-02-11

**Authors:** Woo Kyung Lee, Sumeja Aljic, Patrick Barry, Latha Ganti

**Affiliations:** 1 Emergency Medicine, Coliseum Medical Centers, Macon, USA; 2 Emergency Medicine, Mercer University School of Medicine, Macon, USA; 3 Emergency Medicine, Envision Physician Services, Plantation, USA; 4 Emergency Medicine, University of Central Florida College of Medicine, Orlando, USA

**Keywords:** hyphema

## Abstract

We present a case of an iris sphincter tear with iridodialysis, mydriasis, and hyphema secondary to a traumatic injury from a bungee cord. The correlation between the mechanism of injury and physical exam findings as well as the emergency department evaluation and management are discussed.

## Introduction

Eye trauma is one of the most common causes of preventable trauma with an estimated 2.0 to 2.4 million cases of eye trauma per year in the United States. The five most common causes of eye injury presenting to the emergency department (ED) include being struck by an object, falling, thermal injury, motor vehicle accidents, and environmental causes such as animal or insect bites. Most of these injuries present as superficial corneal injuries, laceration of the eyelid, or bruising of the eye [1-3]. However, other more serious injuries may also occur.

Iris sphincter tears are a common finding in blunt trauma to the anterior eye. Separation between the iris root and ciliary body is referred to as iridodialysis. The sphincter muscles become irreversibly damaged and the subsequent mydriasis causes visual disturbances including glaucoma [4]. Hyphema is another presentation of ocular trauma in which blood pools in the anterior chamber following rupture of eye vessels [5]. We present a case of traumatic iridodialysis associated with hyphema secondary to traumatic injury from a bungee cord.

## Case presentation

A 41-year-old male presented to the emergency department with left eye pain after sustaining a traumatic injury to the left eye from a rusted and hooked bungee cord. He reported additional symptoms of left eye blurry vision, photophobia, redness, swelling, headache, and nausea. He denied loss of consciousness. His vital signs were stable. He has a past medical history of hypertension and bipolar disorder.

**Figure 1:**
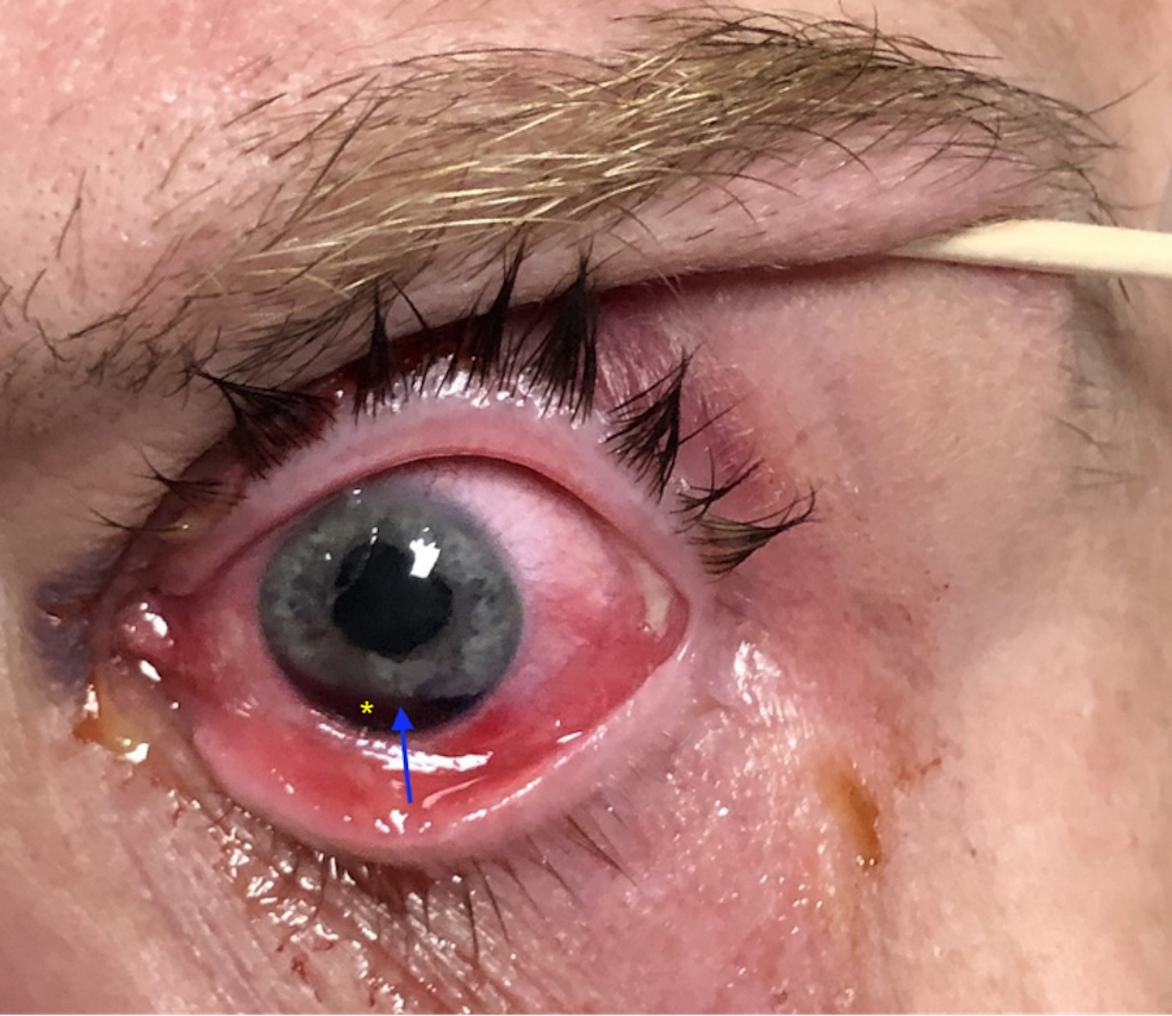
Left eye showing iridodialysis (blue arrow), mydriasis, and hyphema (asterisk).

A focused physical examination of the eyes revealed a dilated left pupil with an irregular shape and absent light reflex. Extraocular movements of the left eye were intact. The patient’s visual acuity was OD: 20/20, OS: 20/800. Intraocular pressure (IOP) was measured three times in each eye and revealed 17/17/17 in the right eye and 17/15/17 in the left eye. Woods lamp test was negative for corneal abrasion or laceration. The Seidel test used to assess leakage of the anterior chamber into the cornea was negative. Hyphema with mydriasis was noted in the left eye (Figure 1). A 0.5 cm abrasion on the medial part of the upper eyelid was also noted. An orbital CT scan did not show any acute changes or eyeball rupture. A traumatic iris sphincter tear with iridodialysis, mydriasis, and hyphema was suspected.

The patient was given tetanus prophylaxis, ondansetron for nausea, and acetaminophen and Ibuprofen for analgesia in the ED. After an ophthalmologic consult, the patient was prescribed prednisolone acetate 1% drop in left eye every one hour and timolol maleate 5 mg by mouth twice daily for seven days. He was also advised to wear an eye shield and elevate his head. He was discharged with instructions to follow up with the ophthalmologist the next day. At three-week follow-up, the patient relayed that the ophthalmologist recommended surgical correction, and was planning the surgery.

## Discussion

During blunt eye trauma, the compressive force on the globe causes an increase in the intraocular pressure. This pressure is dissipated by configurational changes in the elastic layers of the eye such as the cornea, corneoscleral junction, and the iris. However, when the pressure exceeds the tolerability of these elastic layers, there will be a tear or rupture in the weaker parts of the eyeball. This appears to be the case in high-velocity injuries, such as the recoiling of a bungee cord. The force of the trauma displaces the aqueous humor in the anterior chamber posteriorly through the pupil where it is met by the firm resistance of the lens. This force cannot be handled by the iris and the sphincter fibers rupture. The result is persistent mydriasis [4].

Hyphema is a result of blood pooling in the anterior chamber from rupture of blood vessels that supply the iris and ciliary body. Initial management of hyphema includes elevating the head and wearing an eye shield to promote resorption of the blood. Rebleed may occur in the first two to five days after the injury with increased risk if the patient delays medical assessment for longer than 24 hours, a large initial hyphema, an IOP greater than 21 mmHg or an initial visual acuity worse than 20/200.

Hyphemas are graded from 0 to IV, with 0 being no blood in anterior chamber and red cells only seen on slit lamp examination, and grade IV being referred to as an “8-ball” hyphema, where 100% of the anterior chamber is filled with blood. Grade 0 has an almost 100% prognosis for good visual acuity outcome whereas a grade IV would only have a 50% chance. Our patient had a grade I hyphema, which corresponds to <1/3 blood in the anterior chamber and a 90% chance for good prognosis.

In one study of 97 patients, risk factors for poor outcome after traumatic hyphema included: significant risk factors were causality, initial visual acuity, onset of injury, and grade of hyphema [6]. Hyphemas rarely result in hospital admission and can be safely managed in the outpatient setting. Iridodialysis may require surgical correction. Prompt follow-up with ophthalmology is imperative [4].

## Conclusions

Eye trauma is a common emergency department presentation. Prompt assessment should include evaluation of the integrity of globe, visual acuity, depth of the anterior chamber, intraocular pressure, pupil size and function. Patients should be educated that the risk of re-bleed with hyphemas is highest between day 2-5. Activity should be limited. Urgent follow-up with an ophthalmologist is imperative. Depending on the grade of hyphema, surgical intervention may also be necessary.
